# Developing a Behavioral Phenotyping Layer for Artificial Intelligence–Driven Predictive Analytics in a Digital Resiliency Course: Protocol for a Randomized Controlled Trial

**DOI:** 10.2196/73773

**Published:** 2025-08-06

**Authors:** Trevor van Mierlo, Rachel Fournier, Siu Kit Yeung, Sofiia Lahutina

**Affiliations:** 1 Evolution Health Torrance, CA United States; 2 Department of Psychology Chinese University of Hong Kong Hong Kong China; 3 Centrum für Affektive Neurowissenschaften Charité - Universitätsmedizin Berlin Germany

**Keywords:** digital mental health, behavioral economics, engagement, attrition, AI-driven personalization, machine learning, self-guided therapy, digital phenotyping, artificial intelligence

## Abstract

**Background:**

Digital interventions for mental health are pivotal for addressing barriers such as stigma, cost, and accessibility, particularly for underserved populations. While the effectiveness of digital interventions has been established, poor adherence and lack of engagement remain critical factors that undermine efficacy. Millions of individuals will never have access to a trained mental health care practitioner, underscoring the need for highly tailored and engaging self-guided resources. This study builds on a prior study that successfully leveraged behavioral economics (nudges and prompts) to enhance engagement. Expanding on that study, this research will focus on building a foundational dataset of behavioral phenotypes to support artificial intelligence (AI)–driven personalization in digital mental health.

**Objective:**

This 6-arm randomized controlled trial aims to analyze user engagement with randomized tips and to-do lists within a resiliency course tailored for Ukrainian refugees affected by the ongoing humanitarian crisis (Спільна Сила), using the EvolutionHealth.care (V-CC Systems Inc) platform. Insights will inform the development of an AI-based personalization system to optimize engagement and address behavioral health challenges. Secondary objectives include identifying demographic and behavioral predictors of engagement and creating a scalable, culturally sensitive intervention model.

**Methods:**

Participants will be recruited through digital outreach, enrolled anonymously, and randomized into 6 groups to compare combinations of tips, nudges, and to-do lists. Engagement metrics (eg, clicks, completion rates, and session duration) and demographic data (eg, age and gender) will be collected. Statistical analyses will include a comparison between arms and interaction testing to evaluate the effectiveness of each intervention component. Ethical safeguards include institutional review board approval, informed consent, and strict data privacy standards.

**Results:**

This protocol was designed in January 2025. α and β testing of the intervention are scheduled to begin in July 2025, with a soft launch anticipated in August 2025. The experiment will remain active until the sample size requirements are met. Live monitoring and periodic data quality checks will be conducted throughout the study duration.

**Conclusions:**

This trial represents a novel approach to behavioral health research by leveraging randomized experimentation to develop AI-ready behavioral datasets. By targeting an underserved and culturally sensitive population, it contributes critical insights toward scalable, personalized digital mental health interventions. Findings may help inform future digital health efforts that aim to improve engagement, accessibility, and long-term adherence.

**Trial Registration:**

Open Science Framework 34rmg; https://osf.io/34rmg

**International Registered Report Identifier (IRRID):**

PRR1-10.2196/73773

## Introduction

### Background

Self-guided digital mental health interventions play a crucial role in overcoming barriers such as stigma, cost, and accessibility, particularly for underserved populations [1-3]. While their efficacy is well documented in meta-studies [4-7], low adherence and lack of engagement limit their potential impact in publicly available interventions [8-14]. Improving user engagement is therefore essential to realizing the full public health value of these scalable tools.

Optimizing engagement is crucial, as research suggests dose-response relationships; higher engagement is associated with better outcomes such as improvements in mental health [15-17]. This mirrors the commercial world, where higher user engagement results in increased revenue [18,19], and research investigating ways to enhance participation is common.

For example, a 2015 study revealed that LinkedIn (Microsoft Corporation) conducted over 400 controlled engagement experiments per day [20]. The language app Duolingo (Duolingo, Inc) regularly conducts controlled experiments to assess the impact of features to make learning more engaging [21]. Meta (Facebook and Instagram) teaches and technically enables business customers to conduct randomized A-B experiments to optimize ad messaging and conversion [22].

Leveraging similar strategies that commercial platforms use to increase engagement holds the potential to both enhance the user experience in digital mental health and improve overall effectiveness. To that aim, this study builds on prior research on the EvolutionHealth.care (V-CC Systems Inc) platform, which leveraged behavioral economics to tailor user experiences to increase platform engagement [23,24].

For reasons described below, there is a need for further research in optimizing self-guided digital health [25-27], which falls far behind other large-scale commercial platforms that also rely on increasing engagement. Unlike large-scale commercial platforms that rigorously test user experience through ongoing experiments, most digital health platforms lack modern personalization methods. This study aims to address that gap by applying insights from behavioral economics and artificial intelligence (AI) to improve real-world adherence.

### Mental Health Care Professional Deficits

Globally, the World Health Organization (WHO) estimates a deficit of 1.2 million mental health professionals [28]. In the United States, 50.6% of adults with any mental illness received treatment [29], and two-thirds of adults with serious mental health illness received treatment [30].

### The Need to Reframe Digital Health Intervention Reach

**Figure 1:**
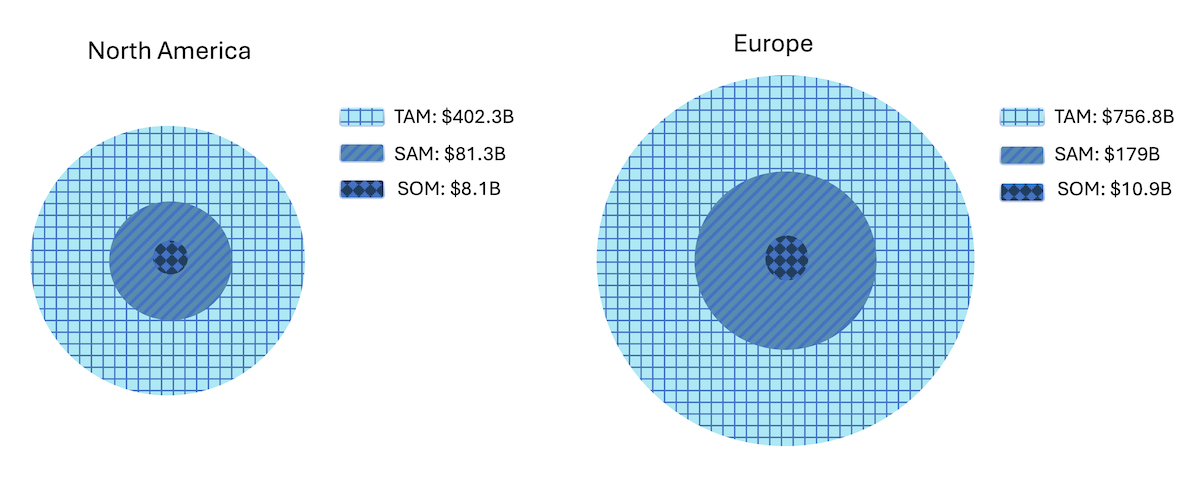
Estimated total addressable market, serviceable available market, and serviceable obtainable market for mental health and substance use in the United States, Canada, and Europe. B: billion; SAM: serviceable available market; SOM: serviceable obtainable market; TAM: total addressable market.

Traditional digital health delivery models are primarily reaching the serviceable obtainable market (SOM)—individuals who are insured, tech-savvy, and already engaged with health care systems. This leaves a significant portion of the population unserved, including those without consistent health care access or digital literacy. Addressing this gap requires intentional design for broader accessibility and lower barriers to entry. The total addressable market (TAM) for mental health interventions is far broader, encompassing displaced individuals, the uninsured, and underserved populations around the world (Figure 1).

The persistent engagement problem suggests that the field has not yet found scalable ways to reach or retain this wider audience. By focusing on open-access, low-barrier design, and tailoring through AI-driven nudges, this study represents an attempt to move beyond the SOM and explore pathways for digital health scale, first reaching the serviceable available market (SAM)—those who have digital devices but are not accessing interventions—and eventually the TAM, the full population of individuals worldwide, including those excluded by platform, policy, or economic barriers.

### Issues With Telehealth Access

Intensified by the COVID-19 pandemic [31,32], digital health interventions have become increasingly common. Investment in mental health treatment, mainly telehealth, has seen significant growth [33,34]. The surge in interest reflects the urgent need to address access gaps identified during public health crises. However, while funding has increased, sustainable implementation remains a major challenge.

Despite billions in funding [35-37], the telehealth sector faces significant challenges. These include market saturation, intense competition, profitability, and workforce shortages. In addition, high costs related to patient acquisition and retention, coupled with regulatory demands and a projected shortage of trained professionals [28,38-40], continue to hinder long-term sustainability. These issues are estimated to extend into the next decade and affect access [41], so there is a need to mitigate these issues with alternative resources.

### AI Models: Large Language Models Versus Predictive Analytics

In digital health, AI has primarily focused on large language models (LLMs). Predictive analytics has fallen behind the use of LLMs, as they require large amounts of AI training data. However, predictive models have the potential to enable personalized user journeys and improve long-term engagement, making them a vital area for future development.

#### LLMs

LLMs are advanced AI systems trained on vast amounts of text data to understand and generate human-like language. In digital health, LLMs power chatbots and virtual assistants that provide patient support, answer health-related queries, and assist health care professionals with information retrieval. These models can engage in natural language conversations, making them valuable tools for enhancing patient education.

#### Predictive Analytics

Predictive analytics involves analyzing current and historical health care data (including behavioral phenotypes) to forecast future outcomes. By leveraging statistical models and machine learning algorithms, health care providers can predict disease progression, patient admissions, and treatment responses. This proactive approach can enable personalized care and tailor user experiences, ultimately improving patient outcomes and operational efficiency.

### Russia’s Invasion of Ukraine and Resulting Displacement

In February 2022, Russia initiated a full-scale invasion of Ukraine, leading to widespread destruction and a significant humanitarian crisis. As a result, millions of Ukrainians have been displaced both internally and internationally, seeking refuge in various countries worldwide.

While exact figures are challenging to determine, Los Angeles has a significant Ukrainian community. The city has seen an influx of Ukrainian refugees since the onset of the war, with many individuals and families seeking to rebuild their lives in the area [42]. Before the 2022 invasion, approximately 60,000 Ukrainian immigrants resided in the state. Following the invasion, an estimated 20,000 or more Ukrainians have arrived, bringing the total to around 80,000 [43]. Major cities such as Los Angeles, San Francisco, and Sacramento have been primary destinations for these new arrivals.

Nationally, the Ukrainian diaspora includes more than 1.1 million individuals, encompassing both immigrants and those of Ukrainian ancestry. As of 2019, there were approximately 355,000 Ukrainian immigrants in the United States. In response to the 2022 invasion, the US government announced plans to accept up to 100,000 Ukrainian refugees. By late 2022, approximately 85,000 Ukrainians had used the “Uniting for Ukraine” program, which allows refugees with an American sponsor to remain in the country for 2 years [44]. By December 2022, President Joe Biden announced that the United States had accepted roughly 221,000 Ukrainian refugees through various programs [45].

Globally, the conflict in Ukraine has led to the displacement of approximately 6.8 million refugees, with nearly 4 million people internally displaced within the country as of November 2024 [46]. European nations have accepted the greatest number of refugees, hosting around 6 million Ukrainians, with Germany now hosting the largest number [47]. Canada has also received a significant number of Ukrainian refugees, with major cities such as Toronto, Montreal, and Winnipeg welcoming thousands of newcomers and implementing resettlement programs to support housing, employment, and cultural integration [48,49]. This mass displacement represents the largest population movement in Europe since World War II [50].

### Behavioral Economics

#### Overview

Behavioral economics leverages psychological experimentation to develop theories about human decision-making. The field has identified a range of biases around how people think and feel. In this study, we will examine the engagement of users with nudges and behavioral prompts in an ad libitum, self-guided digital behavior change course.

Digital interventions can be customized using behavioral economics to correspond with the needs and behaviors associated with specific user types. Since interventions become more appealing and relevant to each user’s unique needs and goals, this personalized approach may improve user engagement. Content and engagement techniques can be adjusted to better match specific goals and motivations by using data on user interactions and behavior trends. Through making digital interventions accessible [51] and culturally acceptable, personalization based on behavioral economics techniques can assist in fulfilling the various demands of different types of populations.

This study’s engagement strategy aligns with the COM-B model of behavior, in which capability (C), opportunity (O), and motivation (M) interact to generate behavior (B) [52]. Directive tips enhance capability through actionable guidance, social proof, and present bias tips increase motivation, and checklists and gamification create opportunity by simplifying follow-through.

#### Nudge Theory

Nudge theory, popularized in the 2008 book *Nudge: Improving Decisions About Health, Wealth, and Happiness* [53], leverages indirect, positive suggestions to influence decision-making and behavior through designs or changes in the decision environment. The approach has been applied in various fields, including finance, education, and public health.

There is a lack of quality research analyzing the use of nudges in digital health. A 2019 scoping review examined the use of nudges in both web-based and real-world settings in physical activity interventions [54]. Of the 35 publications reviewed, 8 were web-based studies. The authors concluded that although nudging may be an effective approach to promote physical activity, there are large gaps in research, and further studies that are explicitly based on nudge insights are needed.

A 2020 editorial in *Personalized Medicine* addressed the meaningful adoption of nudges in digital health [55]. The authors acknowledged that using nudges in digital health interventions is rare and advocated for the use of nudges to promote positive behavior change. This reinforces the relevance of our trial, which aims to rigorously evaluate nudge-based strategies in a digital health setting.

Nudges must be culturally sensitive and adaptable to different cultural backgrounds [56]. This ensures that interventions work and are accepted in many kinds of cultural contexts. Addressing ethical issues, including consent, privacy, and potential biases in nudge design, is essential as nudges become more common in digital health. Reliability in digital health platforms can be strengthened by ensuring transparency and user control over the use of nudges, which we will use in this study [57].

#### Behavioral Prompts

Behavioral prompts are cues specifically designed to encourage a specific task [58]. In this study, we will use 2 types of behavioral prompts anchored in nudge theory: daily tips and a to-do checklist (Table 1). These prompts are intended to support self-guided action, reinforcing positive behavior without requiring direct human intervention.

An effective approach for boosting user involvement in digital health interventions may be offered by analyzing combinations of behavioral prompts that users engage with. Through machine learning techniques, the dataset emerging from this study may provide insight into which and what prompts work best with specific demographics and usage patterns [59]. By customizing interventions to meet the unique needs of every user, this integrated approach might increase the possibility of long-term behavior change and better health outcomes.

**Table 1:** Example nudges and prompts.

Delivery format	Content type	Example from our study
Tip	Directive content	Express yourself by uploading your image!
Tip	Social proof	Many members have similar goals to yours. Reviewing other members’ goals can help you reach your goal.
Tip	Present bias	Feel better sooner by learning from others. Read what others have posted in the community.
Prompt	To-do checklist	Watch the getting started video.
Visual element	Gamification	A progress bar fills as users complete sections of the course.

### Gamification

The use of game-like elements such as progress bars, badges, and rewards has been shown to increase user motivation and adherence in digital health interventions. These strategies leverage principles of intrinsic motivation, offering visual reinforcement and a sense of accomplishment as users complete tasks. When implemented thoughtfully, gamification can help sustain user engagement over time, especially in self-guided programs.

In our previous work, we explored the application of gamified tools to promote physical activity through a social, multiuser platform. This study highlighted the promise and limitations of these techniques in health behavior change [60]. In the current study, gamification is implemented through a dynamic progress bar that visually tracks user progress, reinforcing engagement through motivational feedback, and awarding badges for completing sessions and passing session quizzes.

### Our Prior Work

We have experimented with nudges and prompts in the past. In our previous 3-arm randomized controlled trial (RCT) [24], arm 1 featured a member home page without nudges or prompts. Arm 2 featured a home page with a tip-of-the-day section. Arm 3 featured a home page with a tip-of-the-day section and a to-do checklist.

Control arm members (1788/13,224, 13.52%) completed an average of 1.5 course components. Arm 2 members (865/13,224, 6.54%) clicked on 5% of tips and completed an average of 1.8 course components. Arm 3 members (1914/13,224, 14.47%) clicked on 5% of tips, completed 2.7 of 8 to-do checklist items, and an average of 2.11 course components. Completion rates in arm 2 were greater than those in arm 1 (*z* score=3.37; *P*<.001), and completion rates in arm 3 were greater than those in arm 1 (*z* score=12.23; *P*<.001). Engagement in all 8 components in arm 3 was higher than that in arm 2 (*z* score=1.31; *P*<.001).

Further analysis confirmed that behavioral economics techniques, such as nudges and prompts, significantly enhance engagement in digital health interventions. Specifically, users in the nudge + prompt condition (arm 3) were 93% more likely to engage compared to those in the control group (arm 1), as indicated by an odds ratio of 1.93 (95% CI 1.71-2.17; *P*<.001). This increase in engagement was reflected across multiple platform activities, including self-assessments, goal setting, and content interaction.

However, while these findings validate the effectiveness of nudges and prompts, the study did not explore how individual-level factors shaped these outcomes. Specifically, we did not examine how the intersection of demographic characteristics and cognitive or behavioral phenotypes influenced engagement, if individual engagement followed properties of power laws [61], or if we could leverage economic tools such as the Gini coefficient to plot participation inequality [62].

Understanding these patterns is important for optimizing future interventions for full personalization and maximizing the effectiveness of digital health tools.

### Objective

The primary objective is to analyze user engagement with randomized tips and to-do lists within a resiliency course tailored for Ukrainian refugees affected by the ongoing humanitarian crisis. Insights will inform the development of an AI-based personalization system to optimize engagement and address behavioral health challenges. This approach is designed to improve real-world adherence while maintaining cultural relevance. Secondary objectives include identifying demographic and behavioral predictors of engagement and creating scalable, culturally sensitive nudging [63,64] and intervention models. These insights will also inform future adaptations of the intervention across languages and cultural contexts.

Since there is a paucity of published research in this area [65-67], our unique dataset holds the potential to customize user experiences and transform digital engagement by leveraging behavioral phenotypes and machine learning. Few studies have explored the predictive potential of behavioral cues in real-world digital health environments. This trial is designed to fill that gap and generate data suitable for training scalable AI-driven models.

## Methods

### Digital Health Platform

The digital health platform used in this study is managed by Evolution Health. EvolutionHealth.care is an evidence-based digital health content provider that features courses based on behavior change techniques, including cognitive behavioral therapy (CBT), stages of change, structured relapse prevention, harm reduction, and quizzes based on brief intervention.

The platform offers interactive courses and quizzes for mental health issues, addiction issues, and obesity. It also contains a moderated community based on social cognitive theory.

Limited memberships are available to individuals who register through the organization’s free-to-consumer program. Full memberships are available through white-label instances licensed to employers, insurance companies, employee assistance programs, educational institutions, nonprofit organizations, for-profit health care organizations, and individual therapists.

### The Intervention

Shared Strength (Спільна Сила) is a self-guided interactive behavior change treatment course based on traditional evidence-based treatments (Table 2). It draws from multiple therapeutic frameworks, including CBT, motivational interviewing, and social cognitive theory. The goal is to provide users with a flexible, culturally sensitive, interactive course that supports emotional resilience and behavior change.

Other interactive courses in the Evolution Health platform have been extensively examined in the literature [10,23,24,61,62,68-90]. Shared Strength mirrors its structure and design (Table 3). This consistency enables us to leverage existing platform infrastructure and previous research insights while applying new engagement strategies.

**Table 2:** Theoretical constructs and evidence-based components.

Theoretical construct	Shared Strength (Спільна Сила)
Brief intervention	✓
Cognitive behavioral therapy	✓
Gamification	✓
Health belief model	✓
Motivational interviewing	✓
Social cognitive theory	✓
Targeting and tailoring	✓

**Table 3:** Main course components.

Course component	Shared Strength (Спільна Сила)
Avatar upload	✓
Course completion certificate	✓
Course worksheets	✓
Gamified CBT^a^ course	✓
Getting started video	✓
Goals exercise	✓
Moderated community	✓
Private messaging	✓
Statistics extranet (for corporate clients)	✓
Tailored depression and anxiety test	✓
Therapist extranet	✓

^a^CBT: cognitive behavioral therapy.

### Course Content Overview

Shared Strength (Спільна Сила) contains nine interactive sessions. Each session is designed to foster emotional resiliency using evidence-based strategies drawn from CBT, behavior change theory, and supportive self-help principles. The structure allows participants to move at their own pace while reinforcing learning and self-efficacy. Key session themes include (1) understanding emotional responses to displacement, (2) managing anxiety and low mood, (3) setting personal goals and building hope, (4) practicing mindfulness and grounding techniques, and (5) strengthening connections and community.

Each session includes short readings and interactive tools, such as goal setting, a resiliency diary, to-do checklists, and a summary quiz to reinforce learning. Users progress at their own pace.

### Cultural Adaptation Process, Participatory Design, and Interdisciplinary Collaboration

Following best practice [63,91-93], the Ukrainian version of the Shared Strength course is undergoing cultural adaptation. This process involves collaboration with a bilingual mental health professional and displaced Ukrainian software developers, each bringing insight into the lived experiences of displacement. The adaptation process includes (1) linguistic translation of all English course material, including prompts and tips; (2) contextual revision of examples, tips, and imagery to align with Ukrainian cultural norms and displacement-related stressors; (3) tone and voice refinement to reflect empathy, autonomy, and strength-based messaging in line with Ukrainian cultural communication preferences; (4) technical development and coding by displaced Ukrainian engineers, ensuring both usability and cultural sensitivity; and (5) clinical accuracy: a Ukrainian psychiatrist with experience in delivering CBT-based behavioral interventions and developing digital health tools will review the content.

In addition to linguistic and cultural adaptation, all content is written using plain-language principles to support accessibility for users with varying literacy levels. Usability testing is being conducted with displaced Ukrainian collaborators to ensure the platform is intuitive and accessible for individuals experiencing stress or trauma. This dual approach enhances both comprehension and user trust.

### Recruitment and Participants

Participants will be recruited through partnerships with nonprofit organizations, digital outreach campaigns, and community organizations. Enrollment will occur via the EvolutionHealth.care platform, where participants will be randomized automatically upon sign-up using a computer-generated sequence, ensuring unbiased allocation.

As part of our ethical commitment to accessibility and user privacy, no personal or demographic data will be collected to identify users as displaced or Ukrainian. While this is a limitation, our dissemination model mitigates this concern by partnering with refugee-serving organizations and providing them with custom-branded course instances (eg, “https://YourOrg.EvolutionHealth.care”) or masking their instance with a full URL (eg, “https://YourChosen URL.extension”).

This strategy enables us to reach targeted communities through trusted channels, reducing the likelihood of sample contamination while maintaining an open-access structure that supports real-world scalability and humanitarian distribution. It allows refugee-serving organizations to promote the intervention under their trusted branding. This partnership model combines targeted outreach with the scalability of digital delivery.

### Randomization

**Figure 2:**
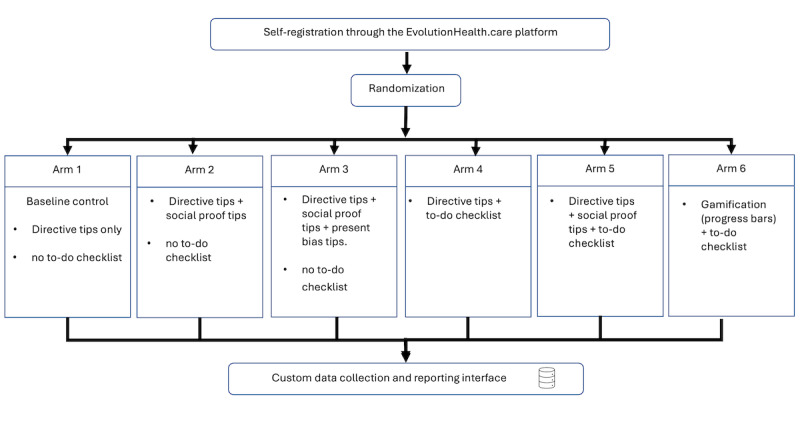
Study flow diagram.

Participants will be randomly assigned to one of the following six arms, as presented in Figure 2:

1. Arm 1 (baseline control): directive tips only and no to-do checklist.
2. Arm 2: directive tips + social proof tips and no to-do checklist.
3. Arm 3: directive tips + social proof tips + present bias tips and no to-do checklist.
4. Arm 4: directive tips + to-do checklist.
5. Arm 5: directive tips + social proof tips + to-do checklist.
6. Arm 6: gamification (progress bars) + to-do checklist.

Participants will be randomly assigned to one of the six experimental arms using computer-generated randomization in a 1:1:1:1:1:1 ratio. Randomization will be conducted at the point of user registration, ensuring equal probability of assignment to any condition. No stratification or blocking will be used, as randomization is expected to produce comparable groups given the planned sample size. Randomization will be logged automatically by the system to ensure reproducibility and allocation integrity.

### Multiarm Trial Rationale

The six study arms were developed to explore the individual and combined effects of behavioral prompts grounded in behavioral economics, to identify which strategies most effectively enhance engagement in a digital resiliency program. Each arm includes a specific configuration of tips, checklists, or gamification elements intended to test distinct motivational mechanisms. This design enables comparative analysis across different engagement strategies while remaining operationally feasible. These components tested include (1) directive tips, grounded in the theory of implementation intentions, which provide straightforward, actionable guidance; (2) social proof tips, which leverage peer effects by referencing the behavior of others to encourage participation; (3) present bias tips, designed to counteract temporal discounting by emphasizing short-term benefits of engagement; (4) to-do checklists, which introduce structured goals and progress-tracking to increase adherence; and (5) gamification, which builds intrinsic motivation through visual indicators of advancement and progress feedback.

While a full factorial design could have explored all possible combinations of these interventions, the current 6-arm structure is a deliberate balance between conceptual rigor and practical feasibility. This structure enables comparative analysis across a diverse set of nudge combinations without creating an unmanageable number of trial arms. It was also informed by operational constraints and the need for meaningful data distribution across arms.

Our study design is also a foundational step toward developing a predictive analytics dataset. The behavioral engagement data collected through the six arms will be used to build baseline models that inform AI-driven personalization strategies. Future iterations of the course will be updated with tailored nudges based on user profiles, and new user data will be used to validate and refine the models. We chose the current design to generate sufficient diversity and volume in behavioral data to create a baseline for our long-term, adaptive research agenda.

### Primary and Secondary Outcomes

The primary outcome is engagement with behavioral prompts, defined as whether a user clicks on a specific tip, type of tip, or checklist item and is directed to the associated course component (refer to Table 3). This outcome reflects the initial behavioral response to a specific nudge. It serves as the basis for understanding which types of prompts are most effective at initiating action within the course. Secondary outcomes reflect broader patterns of use and progression through the course. These include (1) completion of main course components (eg, submitting a goal, completing the depression and anxiety test, completing course sessions, and engaging with other tools defined in Table 3); (2) click rate, defined as the proportion of tips and checklist items clicked; (3) session duration, or the total time spent on the platform; and (4) number of course sessions completed, including badge acquisitions and quiz completions.

### Power and Sample Size

The primary outcome of interest is engagement, measured through click-through rates, session duration, and to-do checklist completion rates across the 6 experimental arms. These metrics allow us to assess how different combinations of prompts and gamification elements affect user behavior. This will help us determine which strategies are most effective for sustaining participation in self-guided programs.

The study is designed to detect small to moderate effect sizes in engagement differences between intervention groups. Expected effect sizes range from Cohen f=0.1 (small) to f=0.25 (moderate). Our goal is to obtain a power of 80% (β=.20) and a significance level of .05 (α) to ensure adequate sensitivity in detecting meaningful differences between groups.

#### Sample Size Calculation for ANOVA

Given that engagement metrics will be compared across 6 arms, a one-way ANOVA test was used to estimate the required sample size. For a small effect size (f=0.1), approximately 86 participants per arm (a total of 516 participants) are required. For a moderate effect size (f=0.25), approximately 28 participants per arm (a total of 168 participants) are required. These calculations assume normality of residuals and homogeneity of variance for ANOVA-based analysis. Chosen effect sizes are based on prior research in digital health engagement studies [94-96]. In our prior RCT [23,24], engagement data were analyzed using mixed-effect logistic regression, which does not rely on these assumptions.

#### Sample Size Calculation for Regression Analysis

Regression analysis will be applied to assess the relationship between engagement and demographic factors (eg, age and gender). This will assist in the identification of predictors of engagement and potential subgroup effects [97]. These analyses are exploratory and may be underpowered. For linear regression, a common rule of thumb suggests 15-20 participants per predictor variable [98,99]. For logistic regression, a minimum of 10 outcome events per predictor variable is typically required [100].

A total of 600 participants will provide sufficient sample size power for multiple predictors while ensuring a balanced distribution across intervention arms. This sample size also accounts for expected variability in engagement behavior across arms and balances analytic rigor with the practical realities of digital recruitment.

#### Sample Size for Repeated Measures

Since engagement data will be collected across multiple time points, generalized estimating equations will be used to account for within-subject correlations. The generalized estimating equation is well-suited for analyzing longitudinal behavioral data in real-world digital environments, allowing more accurate estimation of SEs and robust modeling of repeated engagement behavior. A within-group correlation of ρ=0.3 was assumed, based on prior studies of engagement in digital health. This estimate aligns with findings from longitudinal analysis in behavioral health studies that report moderate within-person correlation of repeated engagement measures [101,102]. A 15% attrition rate was factored into the final sample size, suggesting an adjusted recruitment target of 100 participants per arm to maintain statistical power.

#### Sample Size Justification

To ensure the predictive model has sufficient training data, the study aims to recruit a minimum of 600 participants (100 per arm). This sample size balances feasibility with analytic rigor, ensuring adequate power to detect differences in engagement and to train AI-driven predictive models. Refer to Multimedia Appendix 1 for the study’s CONSORT-SPI (Consolidated Standards of Reporting Trials-Social and Psychological Interventions) checklist [103].

### Data Collection

The Evolution Health platform is equipped with a custom data collection interface and reporting mechanism. Data will be collected for each member who is randomly assigned to the experiment, and all data are self-reported. Information on age and self-identified gender will be collected at registration or during secure sign-on across various white-label instances.

The custom database will also track behavioral engagement with each tip and to-do item randomly presented to users, including whether a tip was displayed, whether it was clicked, and whether the user completed the corresponding course component.

### Engagement Metrics: Operational Definitions

Engagement is defined as follows:

- Click rate is defined as tips or checklist items that users click on.
- Tip interaction is considered “complete” when a user clicks on a tip and completes an exercise.
- Gamification metrics (eg, progress bar status, quiz completion, badge acquisition) will be recorded as binary variables (earned vs not earned).
- Session duration is calculated as the elapsed time (in seconds) between a user logging in and logging out of the platform.

### Ethical Considerations

All data collection policies and procedures adhere to international privacy guidelines, including the General Data Protection Regulation, the US Health Insurance Portability and Accountability Act, where applicable, and the Declaration of Helsinki [104-107]. At registration, all members endorse a checkbox confirming that they consent to have their data used for research purposes and approve the platform’s privacy policy.

The platform does not collect personally identifiable information except a user’s email address, which is required for registration confirmation, retrieval of lost passwords, and two-factor authentication. Email addresses are encrypted and stored in a separate database.

This study is conducted on a self-guided resiliency course for displaced Ukrainian refugees, but it does not measure clinical outcomes. While participants are randomized into 6 intervention arms, the study is classified as an RCT, but it is not an RCT under medical research standards [108]. To clarify, this study adheres to principles of an RCT, where participants are randomly assigned to conditions, but does not fall under medical trial classification as it does not measure health outcomes. The CONSORT-SPI guidelines for social and psychological intervention trials will be followed [103].

The primary outcome variable being tested is course engagement, not wellness or symptom reduction. The study does not evaluate whether participant engagement with course tools improves mental health or reduces emotional stress related to forced displacement. Any potential clinical effects of engagement strategies tested in this study will require investigation in future research.

Since this study does not measure clinical outcomes, it does not fall under ClinicalTrials.gov or the WHO International Clinical Trials Registry Platform requirements. However, to ensure transparency, the trial was registered with Open Science Framework, a widely recognized registry for social science and behavioral health research.

As the study was based on deidentified user data and did not collect medical or clinical measures, it was deemed exempt from further review by Evolution Health’s Institutional Review Board. This exemption is per regulatory standards for minimal-risk research involving nonidentifiable behavioral data.

## Results

This protocol was designed in January 2025. α and β testing of the intervention are scheduled to begin in July 2025, with a soft launch anticipated in August 2025. The experiment will remain active until the sample size requirements are met. Live monitoring and periodic data quality checks will be conducted throughout the study duration.

## Discussion

### Hypothesis

From our previous study, we anticipate higher engagement in arms that include the to-do checklist, particularly when combined with gamification (arm 6) or social proof tips (arm 5) (H1 and H2). Previously, these combinations demonstrated higher click-through and completion rates in earlier research. We expect similar trends in this trial, though results may vary based on population characteristics and platform context.

The study will also contribute to the development of AI models capable of predicting and optimizing user engagement, allowing for the customized delivery of to-do items and tips based on behavioral phenotypes (H2). These models will be trained using the behavioral data collected across study arms. Ultimately, this approach aims to enhance long-term adherence by tailoring interventions to individual patterns of use.

In our previous research on user engagement within the platform’s social network, we observed that engagement patterns followed power law distributions [88]. A follow-up study found that demographic and condition-specific characteristics did not predict social network engagement [89]. However, this study will help determine whether course engagement follows similar power law properties or if age and gender are predictive of engagement outcomes, particularly given the inclusion of session duration and behavioral phenotypes as new analytical factors (H3).

### Expected Results

This study builds on prior RCT findings, extending the use of behavioral economics to underserved, culturally sensitive populations. Unlike existing studies by platforms, our focus on mental health and refugees offers unique contributions to both research and practice. Findings may also advance AI-driven personalization in digital health.

### Strengths and Limitations

A strength of this experiment is that it will be conducted in an ad libitum environment. Unlike most digital health studies, it will not be conducted with a small population in a controlled environment. Further, members will not be aware of the experiment, which will limit participant bias and the Hawthorne effect.

Based on stratified randomization, which will balance demographic factors, the dataset generated will enable predictive analytics for adaptive engagement strategies. Other indication-specific data, such as results and qualifying criteria from the mental health screener [81], have the potential to enrich tailoring. These layers of data will help evolve the platform from static guidance toward real-time personalization.

A limitation of this experiment is that, especially due to the anonymity of members, we have no way of confirming user identity. Registrants may be displaced Ukrainians, individuals reviewing the program based on general interest, researchers, practitioners reviewing the platform for professional use, Evolution Health competitors, industry analysts, or other users who may not be engaging with the platform to help achieve wellness. Sensitivity analyses will be conducted to filter and exclude anomalous user engagement data.

Although engagement correlates with improved intervention outcomes, this study does not measure wellness improvements. Follow-up research will be required to establish clinical efficacy.

### Future Directions

By leveraging randomized experiments and AI, this study provides a blueprint for scalable, culturally sensitive digital health interventions based on demographic characteristics, indication-specific variables, and behavioral phenotypes. This methodology allows for the continuous refinement of engagement strategies over time. The framework can be adapted to support different populations and health indications across diverse settings.

Engagement data from this trial will serve as the foundation for training predictive analytics models that identify which behavioral prompts are most effective for specific user profiles. These models will help personalize the delivery of content in real time, increasing the likelihood of user interaction. Over time, this data-driven approach may significantly improve long-term platform engagement.

As data volume increases (training and validation data), models will be refined and validated using new user cohorts, allowing us to optimize recommendations and nudge strategies based on user behavior, language, and engagement patterns. This iterative refinement process will enhance model accuracy and reduce overfitting. It also enables better generalization across diverse user segments and use cases.

This adaptive approach will also support future deployment of the course in additional languages and for other displaced populations. The underlying platform is designed to be modular, making translation and cultural adaptation straightforward. This scalability is essential for addressing global mental health disparities across diverse regions.

### Conclusions

This trial represents a novel approach to behavioral health research by leveraging randomized experimentation to develop AI-ready behavioral datasets. By targeting an underserved, international, and culturally sensitive population, it contributes critical insights toward scalable, personalized digital mental health interventions. Findings may help inform future digital health efforts that aim to improve engagement, accessibility, and long-term adherence.

## Appendix

Multimedia Appendix 1 CONSORT (Consolidated Standards of Reporting Trials)–Social and Psychological Interventions checklist.

## Abbreviations

AI: artificial intelligence
CBT: cognitive behavioral therapy
LLM: large language model
NAMI: National Alliance on Mental Health
NIMH: National Institute of Mental Health
RCT: randomized controlled trial
SAM: serviceable available market
SOM: serviceable obtainable market
TAM: total addressable market
WHO: World Health Organization

## Data Availability

The datasets generated or analyzed during this study are not publicly available due to the platform’s data privacy policy. Allowing the dataset in this study to be publicly available would violate the platform’s data privacy policy, terms of use, and user agreement. The platform is designed to protect user anonymity, and releasing the full dataset could compromise these commitments. For these reasons, public access to raw data is restricted to protect participant privacy and system integrity. Furthermore, the data emerging from this experiment will be leveraged to train AI models to better understand how to increase and encourage healthy behavior change. Making this data publicly available may have the opposite effect and enable the development of models that can detect and target vulnerable populations. However, the dataset from this study, ongoing engagement data that is continually collected, or other platform datasets can be made available to researchers interested in conducting studies for noncommercial purposes. Researchers must agree to strict data use and privacy terms in line with the platform’s ethical standards. This ensures that data-sharing efforts remain aligned with our core values of participant protection and responsible research. Interested researchers are encouraged to contact Evolution Health.
