# Supplementary material for: Developing a Behavioral Phenotyping Layer for Artificial Intelligence–Driven Predictive Analytics in a Digital Resiliency Course: Protocol for a Randomized Controlled Trial
Source: JMIR Res Protoc. 2025 Aug 6;14:e73773. doi: 10.2196/73773 (PMC12368470; doi:10.2196/73773)
Supplement: Multimedia Appendix 1 [file resprot_v14i1e73773_app1.pdf]

CONSORT-SPI 2018 Checklist

SECTION	ITEM #	CONSORT 2010	CONSORT-SPI 2018	REPORTED ON PAGE #
**TITLE AND ABSTRACT**				
	1a	Identification as a randomised trial in the title§		1
	1b	Structured summary of trial design, methods, results, and conclusions (for specific guidance see CONSORT for Abstracts)§	Refer to CONSORT extension for social and psychological intervention trial abstracts	1
**INTRODUCTION**				
Background and Objectives	2a	Scientific background and explanation of rationale §		2-8
	2b	Specific objectives or hypotheses §	If pre-specified, how the intervention was hypothesied to work	8, 13
**METHODS**				
Trial Design	3a	Describe of trial design (such as parallel, factorial), including allocation ratio §	If the unit of random assignment is not the individual, please refer to CONSORT for Cluster Randomized Trials	10-11
	3b	Important changes to methods after trial commencement (such as eligibility criteria), with reasons		N/A
Participants	4a	Eligibility criteria for participants§	When applicable, eligibility criteria for settings and those delivering the interventions	9
	4b	Settings and locations where the data were collected		12
Interventions	5	The interventions for each group with sufficient details to allow replication, including how and when they are actually administered §		10-11
	5a		Extent to which interventions were actually delivered by providers and taken up by participants as planned	N/A
	5b		Where other informational materials about delivering the intervention can be accessed	N/A

	5c		When applicable, how intervention providers were assigned to each group	9
Outcomes	6a	Completely defined pre-specified outcomes, including how and when they were assessed§		N/A
	6b	Any changes to trial outcomes after the trial commenced, with reasons		N/A
Sample Size	7a	How sample size was determined§		11-12
	7b	When applicable, explanation of any interim analyses and stopping guidelines		N/A
**RANDOMISATION**				
Sequence generation	8a	Method used to generate the random allocation sequence		10-11
	8b	Type of randomisation; detail of any restriction (such as blocking and block size)§		11
Allocation concealment mechanism	9	Mechanism used to implement the random allocation sequence, describing any steps taken to conceal the sequence until interventions were assigned§		11
Implementation	10	Who generated the random allocation sequence, who enrolled participants, and who assigned participants to interventions§		11
Awareness of assignment	11a	Who was aware of intervention assignment after allocation (for example, participants, providers, those assessing outcomes), and how any masking was done		11
	11b	If relevant, description of the similarity of interventions		10-11
Analytical methods	12a	Statistical methods used to compare group outcomes§	How missing data were handled, with details of any imputation method	11-12

	12b	Methods for additional analyses, such as subgroup analyses, adjusted analyses, and process evaluations		N/A
**RESULTS**				
Participant flow (a diagram is strongly recommended)	13a	For each group, the numbers randomly assigned, receiving the intended intervention, and analysed for the outcomes§	Where possible, the number approached, screened, and eligible prior to random assignment, with reasons for non-enrolment	9
	13b	For each group, losses and exclusions after randomisation, together with reasons§		N/A
Recruitment	14a	Dates defining the periods of recruitment and follow-up		N/A
	14b	Why the trial ended or was stopped		N/A
Baseline data	15	A table showing baseline characteristics for each group§	Include socioeconomic variables where applicable	N/A
Numbers analysed	16	For each group, number included in each analysis and whether the analysis was by original assigned groups§		N/A
Outcomes and estimation	17a	For each outcome, results for each group, and the estimated effect size and its precision (such as 95% confidence interval)§	Indicate availability of trial data	14
	17b	For binary outcomes, the presentation of both absolute and relative effect sizes is recommended		N/A
Ancillary analyses	18	Results of any other analyses performed, including subgroup analyses, adjusted analyses, and process evaluations, distinguishing pre-specified from exploratory		N/A
Harms	19	All important harms or unintended effects in each group (for specific guidance see CONSORT for Harms)		N/A
**DISCUSSION**				
Limitations	20	Summarize the main results (including an overview of concepts, themes, and types of evidence available), link to the review questions and objectives, and consider the relevance to key groups.	Trial limitations, addressing sources of potential bias, imprecision, and, if relevant, multiplicity of analyses	13-14

Generalisability	21	Discuss the limitations of the scoping review process.	Generalisability (external validity, applicability) of the trial findings§	13-14
Interpretation	22	Provide a general interpretation of the results with respect to the review questions and objectives, as well as potential implications and/or next steps.	Interpretation consistent with results, balancing benefits and harms, and considering other relevant evidence	13-14
**IMPORTANT INFORMATION**				
Registration	23	Registration number and name of trial registry		Forthcoming
Protocol	24	Where the full trial protocol can be accessed, if available		Forthcoming
Declaration of Interests	25	Sources of funding and other support; role of funders	Declaration of any other potential interests	14
Stakeholder investments	26a		Any involvement of the intervention developer in the design, conduct, analysis, or reporting of the trial	14
	26b		Other stakeholder involvement in trial design, conduct, or analyses	14
	26c		Incentives offered as part of the trial	N/A

This table lists items from the CONSORT 2010 checklist (with some modifications for social and psychological intervention trials) and additional items in the CONSORT-SPI 2018 extension. Empty rows in the 'CONSORT-SPI 2018' column indicate that there is no extension to the CONSORT 2010 item

*We strongly recommended that the CONSORT-SPI 2018 Explanation and Elaboration (E&E) document be reviewed when using the CONSORT-SPI 2018 checklist for important clarifications on each item

§An extension item for cluster trials exists for this CONSORT 2010 item

This checklist is derived from:

- Montgomery, P., Grant, S., Mayo-Wilson, E., Macdonald, G., Michie, S., Hopewell, S., & Moher, D. (2018). Reporting randomised trials of social and psychological interventions: the CONSORT-SPI 2018 Extension. *Trials*, *19*(1), 407.
- Grant, S., Mayo-Wilson, E., Montgomery, P., Macdonald, G., Michie, S., Hopewell, S., & Moher, D. (2018). CONSORT-SPI 2018 Explanation and Elaboration: guidance for reporting social and psychological intervention trials. *Trials*, *19*(1), 406.

- Schulz, K. F., Altman, D. G., & Moher, D. (2010). CONSORT 2010 Statement: updated guidelines for reporting parallel group randomised trials. *BMJ*, *340*, c332.

Montgomery 2018 and Grant 2018 were distributed under the terms of the Creative Commons Attribution 4.0 International License (http://creativecommons.org/licenses/by/4.0/). Schulz 2010 was distributed under the terms of a Creative Commons Attribution Non-commercial License (https://creativecommons.org/licenses/by-nc/2.0/). We have revised the checklists as published to include an extra column for “reporting on page #”.
